# Analysis of Wilms Tumour Epidemiology, Clinicopathological Features and Treatment Outcomes in 84 Moroccan Patients

**DOI:** 10.1002/cnr2.2158

**Published:** 2024-11-07

**Authors:** Sara Benlhachemi, Mohammed Khattab, Kenza Hattoufi, Redouane Abouqal, Saber Boutayeb, Elmostafa El Fahime

**Affiliations:** ^1^ Neuroscience and Neurogenetics Research Team (ERNN), Faculty of Medicine and Pharmacy Mohammed V University Rabat Morocco; ^2^ Molecular Biology and Functional Genomics Platform National Centre for Scientific and Technical Research Rabat Morocco; ^3^ Department of Paediatric Haematology and Oncology Abulcasis International University of Health Sciences Rabat Morocco; ^4^ Department of Neonatology and Nutrition University Hospital Centre IBN SINA Rabat Morocco; ^5^ Research Team on Health and Nutrition of Mother and Child, Faculty of Medicine and Pharmacy Mohammed V University Rabat Morocco; ^6^ Biostatistics Laboratory, Clinical Epidemiology Research, Faculty of Medicine and Pharmacy Mohammed V University Rabat Morocco; ^7^ Medical Oncology Department National Institute of Oncology, Mohammed V University Rabat Morocco; ^8^ Mohamed VI Centre for Research and Innovation (CM6RI) Rabat Morocco

**Keywords:** clinicopathology, histopathology, SIOP protocol, treatment, Wilms tumour

## Abstract

**Background:**

Wilms tumour (WT), the second most reported childhood cancer in Morocco, is a malignant kidney tumour that affects children under 15 years old. Prognosis has improved with the adoption of multimodal treatment. However, data on WT in Morocco remain limited.

**Aims:**

This study aims to comprehensively describe and analyse the epidemiological, clinicopathological features and treatment outcomes of WT in Moroccan patients, including treatment response and recurrence rates.

**Methods and Results:**

A retrospective study involved 84 children under 15 years with WT, treated according to the SIOP protocol and followed at the Paediatric Haematology and Oncology Centre at Children's hospital of Rabat, between January 2014 and February 2018. The median age of participants was 36 months, with a male/female sex ratio of 0.79. Abdominal mass was the primary concern in 55 cases (66%). Five patients (6%) had bilateral WT. Metastatic WT occurred in 21 cases (25%). Stage III was predominant in 33 cases (43%). Twenty cases (26%) had high‐risk WT, and IVC tumour thrombus was observed in 12 cases (14%). WT histotype correlated significantly with both sex and tumour localisation (*p* values of 0.040 and 0.013, respectively). Age correlated significantly with WT extension, overall stage and SIOP histology risk grades (*p* values of 0.003, 0.003 and 0.045, respectively). Overall stage was statistically related to the occurrence of IVC tumour thrombus (*p* = 0.002). Over a 5‐year span post‐nephrectomy, complete remission was achieved in 63 patients (75%), partial remission in one patient (1%), while 19 patients (23%) died and one patient (1%) relapsed.

**Conclusion:**

These findings are encouraging for a developing country. However, the elevated rates of Stages III and IVC thrombus in this series are still high, primarily attributed to delays in diagnosis and treatment and the limited number of paediatric haematology and oncology units at the time of the study. Nevertheless, further multicentric research is warranted to enrich Moroccan data and establish a national register for WT cases.

**Abbreviations**
ACT: d‐actinomycin
ASA: American Society of Anaesthesiologists
BWS: Beckwith–Wiedemann syndrome
CBC: complete blood count
CBEU: cytobacteriological exam of urine
CT: computed tomography
Hb: haemoglobin
HVA: homovanillic acid
IVC: inferior vena cava
MRI: magnetic resonance imaging
NB: neuroblastoma
PLT: platelets
PMN: polymorphonuclear neutrophils
SIOP: International Society of Paediatric Oncology
VCR: vincristine
VMA: vanillylmandelic acid
WBC: white blood count
WT: Wilms tumour

## 1 Introduction

Wilms tumour (WT) or nephroblastoma, was initially documented by Dr. Max Wilms in 1899 [1]. This tumour is embryonic in nature and originates from the malignant transformation of renal progenitor cells that have preserved their embryonic differentiation potential. These cells are considered precursors of WT and are found in 25% to 40% of patients [2]. The incidence of WT is highest among African children, estimated at 16.5 per million, followed by Caucasian, and then Asian children [3, 4]. In Morocco, it ranks as the second most reported paediatric cancer, following acute lymphoblastic leukaemia [5]. In Europe and North America, the annual incidence rate stands at 8.2 cases per 1 million children under the age of 15 [6], typically affecting children between 1 and 5 years old [7].

Currently, a multimodal approach comprising surgical resection, preoperative and/or postoperative chemotherapy and in some cases x‐ray treatment succeeds an overall survival (OS) exceeding 90% for localised WT, 75% for metastatic WT and 50% in the case of WT recurrence [8, 9].

In Morocco, there are six paediatric haematology and oncology units (in Rabat, Casablanca, Marrakech, Fes and Oujda cities) [10], in which WT is managed following the International Society of Paediatric Oncology (SIOP) protocol. However, there is a limited amount of comprehensive data regarding the epidemiology, clinical features and disease progression, presenting a major challenge for Moroccan paediatric oncologists. Through this retrospective study, we aim to elucidate the clinical and pathological characteristics of WT and the outcomes of the multimodal treatment employed in our institution, namely, the Paediatric Haematology and Oncology Centre within the Children's Hospital of Rabat.

## 2 Materials and Methods

### 2.1 Study Design

The present study adopted a retrospective, descriptive and exploratory methodology, conducted at the Paediatric Haematology and Oncology Centre at the Children's Hospital in Rabat, covering the period from 1 January 2014 to 28 February 2018.

### 2.2 Patient Enrolment

#### 2.2.1 Inclusion Criteria

Patients with WT were recruited by fulfilling the following inclusion criteria: (i) female or male patients, (ii) under 15 years of age, (iii) with confirmed WT (iv) diagnosed and treated at our institution. All of the included patients underwent physical exam, imaging exams (abdominal ultrasound and CT scan, chest x‐ray), complete blood count (CBC), urea, serum creatinine, vanillylmandelic acid (VMA), homovanillic acid (HVA) and urinary dopamine where available.

#### 2.2.2 Exclusion Criteria

**FIGURE 1:**
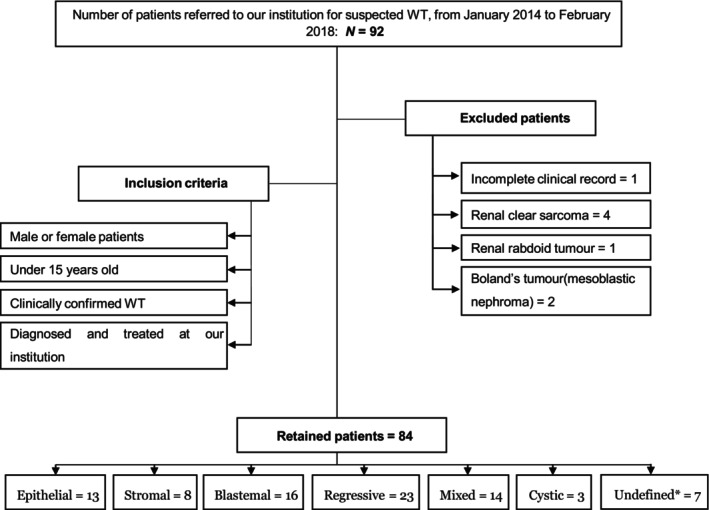
Flow diagram of patient selection. Asterisk indicates that the undefined group refers to patients who died prior nephrectomy and anatomopathological analysis of the operative specimen.

Conversely, patients were ineligible for inclusion if (i) their medical records were incomplete or (ii) they had other kidney tumours, such as renal clear sarcoma, renal rabdoid tumour, or mesoblastic nephroma (Boland's tumour) (Figure 1).

### 2.3 Data Collection

Data collection involved a comprehensive examination of clinical records using patient follow‐up template. The latter encompassed the following sections: *epidemiological data* (including sex, age, clinical history and comorbidities); *clinical features* (medical complaint, comprising symptoms, tumour characteristics, metastatic extensions and anatomopathological findings); *treatment regimen*; *clinical evaluation* and *treatment outcomes* (including complete remission, relapse or mortality).

### 2.4 Statistical Assessment

We carried out the statistical analysis using Jamovi biostatistical software (Version 2.3.28) [11]. We initiated the analysis with a descriptive exploration. Qualitative variables were represented as counts and percentages. Symmetrically distributed quantitative variables were represented as means and standard deviations, while asymmetrically distributed quantitative variables were represented using medians and quartiles. The normality of quantitative variables was assessed using Shapiro–Wilk test.

Following this, we proceeded to perform an inquiry into the association between different variables, using either Pearson's chi‐square test or Fisher's exact test to compare the frequencies of categorical variables. A *p* value below 0.05 was considered statistically significant.

We excluded the cystic and bilateral groups from the association analysis due to their small patient counts, three and five, respectively. This aimed to ensure that each group had an adequate number of cases, hence reducing the possibility for bias and ensuring the reliability of our analysis.

The age categories include infants aged from 0 to 24 months, early childhood spanning from 2 to 5 years and middle childhood beginning at 6 years and beyond.

### 2.5 Ethical Considerations

The research protocol received approval from the Ethics Committee for Biomedical Research of the faculty of Medicine and Pharmacy, Mohamed V University of Rabat, Morocco; with reference number CERB 12/22. Informed consent was obtained from the parents or legal guardians of the included children affected with WT. The data processing procedures were carried out with careful attention to principles of security and confidentiality. To ensure total anonymity, a unique code was assigned to each patient, therefore preventing any possibility of direct or indirect identification.

### 2.6 SIOP Protocol for WT Management

**FIGURE 2:**
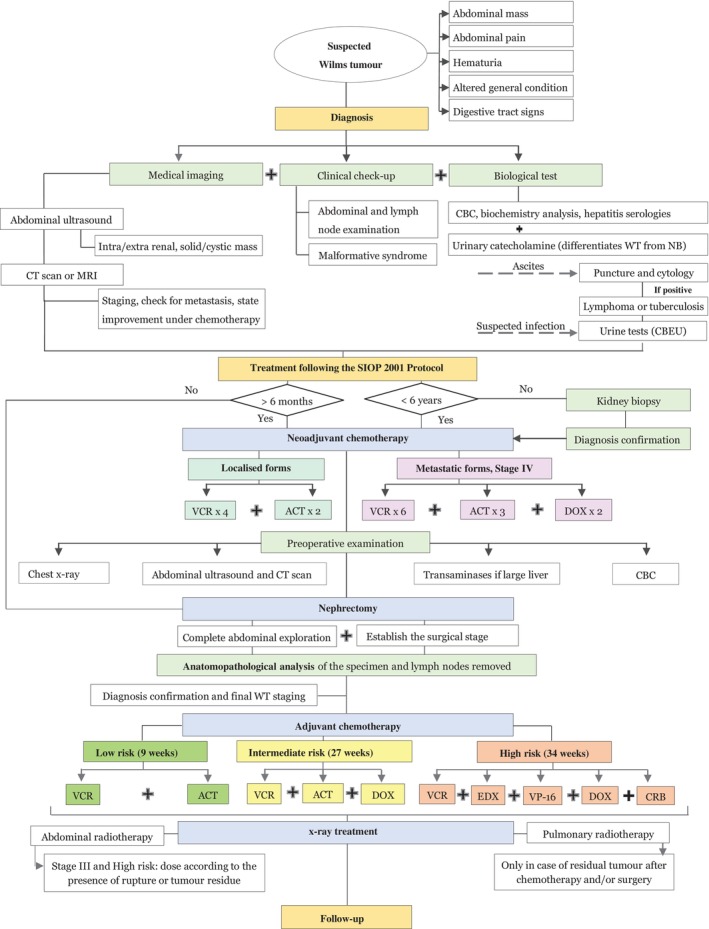
WT management according to the SIOP 2001 protocol followed by the Paediatric Haematology and Oncology Centre of Rabat Children's Hospital. ACT, actinomycine‐D; CBC, complete blood count; CBEU, cytobacteriological exam of urine; CRB, Carboplatine; CT, computed tomography; DOX, doxorubicine; EDX, Endoxan; MRI, magnetic resonance imaging; NB, neuroblastoma; VCR, vincristine; VP‐16, Etoposide; WT, Wilms tumour.

The SIOP protocol is a multidisciplinary approach involving a diverse medical team, including oncologists, paediatricians, radiologists, pathologists, radiotherapists, biologists, paediatric surgeons, haematologists, nurses and others. Figure 2 illustrates the SIOP 2001 protocol as followed by our institution.

Diagnosis and monitoring of patients undergoing therapy rely primarily on clinical, biological and radiological findings. Imaging is crucial in surgical planning; abdominal ultrasound and computed tomography (CT) scan or magnetic resonance imaging (MRI) establish a presumptive WT diagnosis [6], chest x‐ray looks for lung or bone metastases and thoracic CT scan confirms and provides details about lung nodes size and location [3]. Routine blood tests include: CBC to look for anaemia or neutropenia, as well as biochemistry analysis to assess renal and liver function [6]. In some cases, urinary catecholamine measurements are conducted to distinguish WT from neuroblastoma (NB), another childhood cancer most often presenting with an abdominal mass [1, 12].

Preoperative chemotherapy is administered weekly, in order to reduce tumour's size prior to surgical intervention. For localised forms, it involves the use of Vincristine (VCR) and d‐actinomycin (ACT), and for metastatic forms, it uses VCR, ACT and Doxorubicin (DOX). Infants less than 6 months of age are treated with primary nephrectomy.

Anatomopathological analysis is performed on the excisional specimen which is usually the affected kidney with the surrounding lymph nodes, in order to distinguish WT from non‐WT and to define the histological subtype and WT stage [13]. Biopsies are often reserved for cases where imaging is not sufficient to establish a definite diagnosis, or when the patient is over 6 years old.

Treatment for WT varies based on the tumour staging and histological features of the patient. It may include nephrectomy, chemotherapy and radiation therapy. Postoperative chemotherapy is then given to all WTs to reduce the likelihood of tumour recurrence. Early WT stages receive VCR and ACT, while advanced stages receive DOX, Endoxan (EDX), Etoposide (VP‐16) and Carboplatin (CRB). Typically, patients in more advanced stages (Stages III and IV) or those with infiltrated surgical margins or renal vein thrombus undergo a risk‐adjusted combination of postoperative chemotherapy and x‐ray treatment [6, 14].

Follow‐up visits are usually scheduled every 3 months for 2 years after diagnosis, then every 6 months for another 2 years, then once a year. They may include abdominal ultrasound, chest x‐ray and/or CT scan + MRI for any doubt of relapse, metastasis, as well as kidney, liver, heart and auditory function tests to detect any long‐ or short‐term toxicity.

## 3 Results

Table 1 presents a comprehensive report of the findings observed among the 84 patients with WT included in our study.

**TABLE 1:** Descriptive summary of the main results.

Epidemiological profile of patients with WT	
Sex (*N* = 84)	*n* (%)
Female	47 (56)
Male	37 (44)
Age at diagnosis (months), Median 50 [25–75]	36 [23–59]
Age ranges (*N* = 84)	
[0–24 months]	22 (26)
[2–5 years]	47 (56)
≥ 6 years	15 (18)
Presence of family history (*N* = 84)	24 (28)
Cancer	22 (26)
Haemopathy	4 (5)
Malformative syndrome	1 (1)
Abnormalities (*N* = 84)	10 (12)
Major clinical abnormalities	2 (20)
Beckwith–Wiedemann syndrome	1 (50)
Hypertelorism	1 (50)
Minor clinical abnormalities	8 (80)
Inguinal hernia	3 (37)
Hypospadias	1 (13)
Undescended testis	2 (24)
Bronchogenic cyst	1 (13)
Hemihypertrophy of the right side	1 (13)
Parental consanguinity (*N* = 84)	19 (23)
WT type (*N* = 84)	
Sporadic	81 (98)
Hereditary	2 (2)

Clinical profile of patients with WT	
Medical complaint (*N* = 83)	
Abdominal mass	55 (66)
Abdomen pain	8 (10)
Macroscopic haematuria	12 (14)
Diagnostic lead time (days) (*N* = 82), median 50 [25–75]	21 [15–60]
<1 month	43 (53)
[1–3 months]	28 (34)
[4–6 months]	7 (8)
[7–9 months]	4 (4)
Digestive symptoms	83 (99)
Abdominal mass or swelling	81 (96)
Abdominal pain	30 (36)
Vomiting	14 (17)
Urinary tract symptoms	16 (19)
Gross haematuria	14 (16)
Microscopic haematuria	2 (2)
Varicocele	1 (1)

Extension workup (ultrasound, CT scan, MRI)	
Tumour localization	
Left kidney	46 (55)
Right kidney	33 (39)
Bilateral	5 (6)
Unilateral tumour size (cm^3^)	
Left kidney, median 50 [25–75]	546 [368–721]
Right kidney, median 50 [25–75]	685 [548–1084]
Tumour features (*n* = 82)	
Solid	50 (61)
Cystic	10 (12)
Mixed	22 (27)
Metastatic extension	21 (25)
Oligometastasis	14 (67)
Polymetastasis	7 (33)
Lung metastasis	17 (81)
Liver metastasis	6 (29)
Node metastasis	7 (33)
Bone metastasis	2 (9)
Effusion	16 (19)
Peritoneal	11 (69)
Pleural	3 (19)
Peritoneal and pleural	2 (12)
IVC tumour thrombus	12 (14)
Renal vein tumour thrombus	9 (12)
Tumour rupture	11 (13)

Biological check‐up	
Anaemia (*n* = 79)	44 (56)
Hb, g/dL (*n* = 78), mean ± SD	10 ± 2
Neutropenia	2 (3)
PMN, mm^3^ (*n* = 72), median 50 [25–75]	4890 [3760–6443]
Leukopenia (*n* = 74)	5 (7)
WBC, mm^3^ (*n* = 74), median 50 [25–75]	10 015 [8178–12 883]
Thrombocytopenia	1 (1)
Thrombocytosis	15 (19)
PLT, mm^3^ (*n* = 78), median 50 [25–75]	361 000 [289 250–462 750]
Catecholamine urine test (*n* = 18)	
VMA, mg/L, mean ± SD	4.86 ± 2.67
HVA, mg/L, median 50 [25–75]	8.1 [6.0–13.1]
Dopamine, μg/g, median 50 [25–75]	636 [466–896]
Urine test (*n* = 69)	
Urea, g/L, median 50 [25–75]	0.18 [0.15–0.24]
Creatinine, mg/L, median 50 [25–75]	4.10 [3.60–4.60]

WT staging and histological subtypes (*n* = 77)	
Epithelial	13 (17)
Stromal	8 (10)
Blastemal	16 (21)
Regressive	23 (30)
Mixed	14 (18)
Cystic partially differentiated	3 (4)
SIOP histology risk grades	
Low	3 (4)
Intermediate	54 (70)
High	20 (26)
Anaplasia (unfavourable histology)	4 (7)
Nephrogenic rest	13 (17)
Nephroblastomatosis	9 (12)
Local WT stage	
Stage I	14 (18)
Stage II	30 (39)
Stage III	33 (43)
Overall WT stage (*n* = 80)	
Stage I	13 (16)
Stage II	20 (25)
Stage III	21 (27)
Stage IV	20 (25)
Stage V	5 (6)

Treatment regimen profile	
Treatment delay in days (*n* = 80), median 50 [25–75]	2 [1–4]
Preoperative chemotherapy	80 (95)
Nephrectomy	77 (92)
Primary nephrectomy	4 (5)
Nephrectomy after chemotherapy	74 (95)
ASA score (*n* = 62)	
ASA I	47 (76)
ASA II	15 (24)
Postoperative chemotherapy	76 (98.7)
X‐ray treatment	38 (49)
Total treatment duration in days, median 50 [25–75]	248 [222–289]

Treatment outcomes (N = 84)	
Median follow‐up (years)	5.55 [2.5–7.1]
Relapse	13 (15)
Local relapse	6 (46)
Metastatic relapse	5 (39)
Local and metastatic relapse	2 (15)
Evolution after relapse (*n* = 13)	
Remission after relapse	5 (38.5)
Death after relapse	8 (61.5)
Progress after 5 years	
Death	19 (23)
Continuous complete remission	63 (75)
Partial remission	1 (1)
Relapse	1 (1)

*Note:* The values are represented as counts and percentages within columns, *n* (%). The normal range for dopamine is below 1300 μg per gram of creatinine.

Abbreviations: ASA, American Society of Anaesthesiologists; CT, computed tomography; Hb, haemoglobin; HVA, homovanillic acid; IVC, inferior vena cava; MRI, magnetic resonance imaging; PLT, platelets; PMN, polymorphonuclear neutrophils; SD, standard deviation; VMA, vanillylmandelic acid; WBC, white blood count; WT, Wilms tumour.

### 3.1 Epidemiological Profile of Patients With WT

The age of our patients ranged from 3.7 months to 10.1 years, with a median age of 36 [23–59] months. Notably, the age group most impacted was between 2 and 5 years, accounting for 56% of cases. There was a slight female predominance, with 47 females (56%) and 37 males (44%), resulting in 0.79 male/female sex ratio. WT preponderated in children from urban areas in 51 cases (65%).

The median delay between the onset of clinical manifestations and the first consultation was 21 [15–60] days. Notably, 53% of the patients sought medical attention within the initial month. Nevertheless, in 70 cases (96%), parents noticed the clinical signs fortuitously. Out of the 24 patients (28%) with a family history, 22 (26%) had a family history of cancer, four (5%) had a history of haematological disorders and one (1%) had a sibling with Down syndrome. Whereas 14 patients (17%) had a personal history of anaemia, prematurity, speech delay and other conditions (each occurring in one or two cases). Parental consanguinity was recorded in 19 patients (23%). While two female first cousins had hereditary WT, 81 cases (98%) were sporadic. There was also a single adopted child whom the family medical history remained unknown. Ten patients (12%) presented with clinical abnormalities, of whom two had major abnormalities, one patient with Beckwith–Wiedemann syndrome (BWS) and the other with hypertelorism. The remaining eight patients had minor abnormalities, including inguinal hernia (in three cases), undescended testicles (in two cases), hypospadias, bronchogenic cysts and hemihypertrophy of the right side (each occurring in one case).

### 3.2 Clinical Profile of Patients With WT

Abdominal mass was the most frequent medical complaint with 55 affected patients (66%). The clinical examination revealed 46 patients (55%) with general clinical signs, including fever in 24 patients (29%), weight loss in 20 cases (24%) and asthenia in 14 cases (16%). Digestive symptoms were prevalent in 83 patients (99%), with abdominal mass being the most common sign in 81 cases (96%). In 16 patients (19%), a urinary sign was manifested, either in the form of gross haematuria in 14 cases (16%) or microscopic haematuria in two cases (2%). Skin lesions appeared in two cases (2%), characterised by light brown spots in one patient and slate‐coloured spots in the other.

**FIGURE 3:**
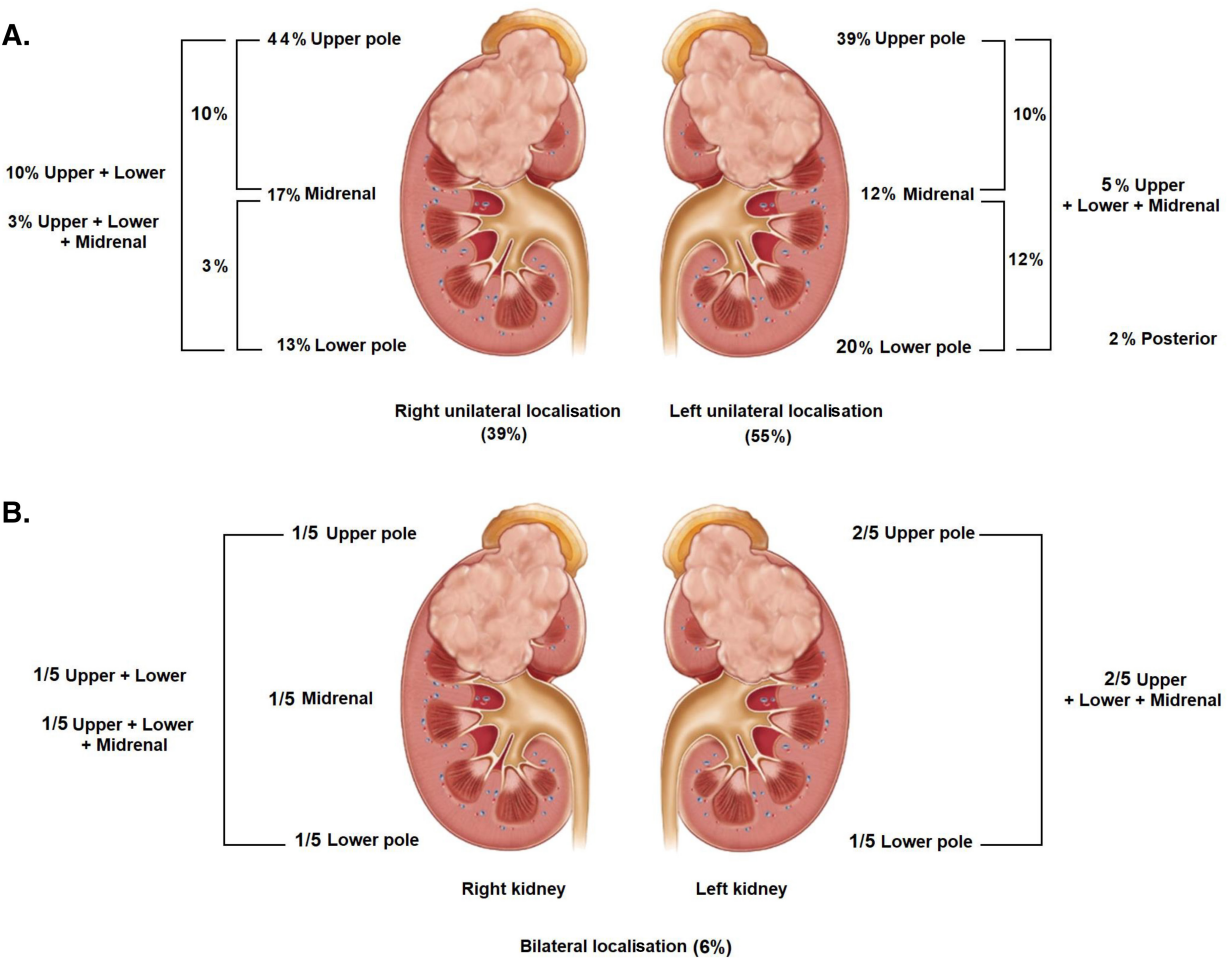
Frequency of WT occurrence across the various segments of the kidneys. (A) Unilateral WT (*N* = 79). (B) Bilateral WT (*N* = 5).

All patients underwent abdominal ultrasound and CT scan, as well as chest x‐ray. Only patients with abnormal chest x‐ray underwent chest CT scans, totalling an estimated 17 patients. Abdominal ultrasound revealed unilateral WT in 79 cases (94%) (Figure 3A), and bilateral WT in five cases (6%) (Figure 3B). Tumour thrombus involving the inferior vena cava (IVC) was observed in 12 cases (14%), while tumour thrombus involving renal vein was noted in nine patients (12%). Tumour rupture was identified in 11 cases (13%). Moreover, the tumour was solid in 50 cases (61%), cystic in 10 cases (12%) and mixed in 22 cases (27%). WT was metastatic in 21 cases (25%); it was oligometastatic (involving one metastatic site) in 14 cases (67% of metastatic cases) and polymetastatic (involving multiple metastatic sites) in seven cases (33% of metastatic cases). Abdominal CT scan revealed liver metastases in six patients (29% of metastatic cases), characterised by hypodense lesions in the liver segments. The frontal chest x‐ray showed pulmonary nodes in favour of lung metastases in 17 patients (81% of metastatic cases).

The initial biological work‐up performed in all our patients, showed anaemia in 44 (56%) cases, and thrombocytosis in 15 (19%) cases. Urinary catecholamine testing was performed in only 18 cases, primarily when the tumour involved the adrenal gland and the upper pole of the kidney, the mean value of VMA was 4.86 ± 2.67 mg/L and the median value of HVA was 8.1 [6.0–13.1] mg/L. All patients exhibited VMA and HVA values within normal ranges, adhering to the reference ranges for VMA <19.28 mg/L and HVA <27.4 mg/L.

### 3.3 WT Staging and Histological Subtypes

**FIGURE 4:**
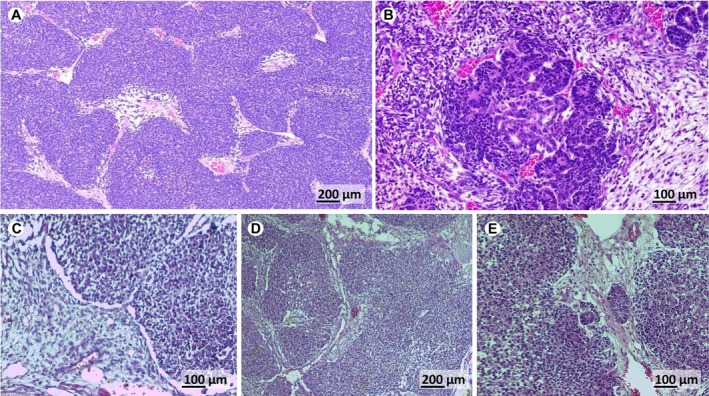
Histological patterns of Wilms tumour (WT). (A) Blastemal components: Dense cellular organisation and small, undifferentiated cells arranged in compact sheets with high nuclear‐to‐cytoplasmic ratios (magnification ×10, scale bar = 200 μm). (B) Mixed WT: heterogeneous mix of primitive mesenchyme, blastemal and epithelial components forming immature tubular structures, indicating early differentiation (magnification ×20, scale bar = 100 μm). (C) Blastema and primitive mesenchyme: the blastemal cells appear as densely packed, undifferentiated cells, whereas the mesenchymal component is less dense and may show early signs of differentiation into fibrous tissue (magnification × 20, scale bar = 100 μm). (D) Rests of blastemal cells: isolated clusters of undifferentiated blastemal cells scattered within the stromal background, suggesting minimal differentiation (magnification × 10, scale bar = 200 μm). (E) Rests of blastemal and epithelial differentiation: regions where blastemal cells coexist with areas of epithelial differentiation forming primitive tubules, indicating early epithelial organisation (magnification ×20, scale bar = 100 μm) (images from the Pathology laboratory at Ibn Sina University Hospital Centre in Rabat).

Out of the 77 patients who underwent nephrectomy, 14 cases (18%) were categorised as local Stage I, 30 patients (39%) as local Stage II and 33 patients (43%) as local Stage III. The regressive histologic subtype was the most dominant, accounting for 23 cases (30%), followed by the blastemal type in 16 cases (21%) and the mixed type in 14 cases (18%) (Figure 4). This resulted in a low SIOP histology risk grade in three cases (4%), intermediate risk in 54 cases (70%) and high risk in 20 cases (26%).

### 3.4 Treatment Regimen Profile

The time interval between diagnosis and the initiation of the treatment ranged from 0 to 31 days, with a median delay of 2 [1–4] days for the completion of the abdominal ultrasound scan and extension assessment. Preoperative chemotherapy was administered to 80 patients (95%), while the remaining four patients, all aged less than 6 months, underwent primary nephrectomy. Seventy‐seven patients (92%) underwent nephrectomy, with 59 patients undergoing lymph node sampling during the surgical operation. Seventy‐six patients (98.7%) undertook postoperative chemotherapy, and 38 patients (49%) received x‐ray treatment.

### 3.5 Treatment Outcomes

During the follow‐up period between 2014 and 2023, the median follow‐up was 5.55 [2.5–7.1] years, with a minimum of 23 days and a maximum of 9.1 years. The 5‐year prognosis revealed complete remission in 63 patients (75%). One patient (1%) remained in partial remission due the persistence of a few nodes in the contralateral kidney. One patient (1%) relapsed after 7 years of remission. Nineteen patients (23%) passed away. Notably, seven patients died before undergoing nephrectomy, due to either tumour progression or chemotherapy‐related toxicity. More specifically, several complications were observed before death, including cardiorespiratory arrest during anaesthetic induction, an IVC tumour thrombus, toxicity (severe infection during febrile neutropenia), brain metastasis, severe malnutrition accompanied by tumour progression, each occurring in one patient, as well as preoperative tumour rupture in two patients. Thirteen cases (15%) had relapses during or after the end of treatment, with five achieving complete remission, while eight passed away.

### 3.6 Assessment of Associations

#### 3.6.1 Association of Histotypes and WT Characteristics and Progression

Table 2 displays the findings of an association analysis conducted to examine the relationship between different WT histotypes and various features of WT. A statistically significant association was observed between WT histotypes and sex (*p* = 0.040). The prevalence of regressive and blastemal types was higher among female patients, accounting for 70% and 81%, respectively. Conversely, the epithelial and mixed types were more often seen in male patients including 62% and 64%, respectively. Similarly, WT histotypes showed a strong association with WT localisation (*p* = 0.013). Specifically, the epithelial and blastemal types were shown to be more abundant in left‐sided WTs, accounting for 92% and 56%, respectively. In contrast, the mixed type had a higher prevalence in right‐sided WTs, accounting for 64%.

**TABLE 2:** Comparison between WT histotypes and WT types and evolution.

	Epithelial (*N* = 13)	Stromal (*N* = 8)	Regressive (*N* = 23)	Blastemal (*N* = 16)	Mixed (*N* = 14)	*p*
Sex						0.040
Female	5 (38)	4 (50)	16 (70)	13 (81)	5 (36)	
Male	8 (62)	4 (50)	7 (30)	3 (19)	9 (64)	
Age range						0.657
< 2 years	4 (31)	2 (25)	2 (9)	3 (19)	4 (29)	
[2–5 years]	7 (54)	6 (75)	15 (65)	9 (56)	7 (50)	
≥ 6 years	2 (15)	0 (0)	6 (26)	4 (25)	3 (21)	
Metastasis						0.411
Absence	11 (85)	5 (63)	14 (61)	12 (75)	12 (86)	
Presence	2 (15)	3 (37)	9 (39)	4 (25)	2 (14)	
Tumour localisation						0.013
Right kidney	1 (8)	3 (37)	12 (52)	4 (25)	9 (64)	
Left kidney	12 (92)	5 (63)	10 (44)	9 (56)	5 (36)	
Bilateral	0 (0)	0 (0)	1 (4)	3 (19)	0 (0)	
Local stage						0.307
Stage I	3 (23)	1 (12)	2 (9)	3 (19)	4 (28)	
Stage II	7 (54)	1 (12)	8 (35)	6 (37)	6 (44)	
Stage III	3 (23)	6 (76)	13 (56)	7 (44)	4 (28)	
Relapse	1 (8)	2 (25)	5 (22)	3 (19)	1 (7)	0.635
Progress after 5 years from nephrectomy						0.433
Death	2 (15)	0 (0)	4 (18)	5 (31)	1 (7)	
Complete remission	11 (85)	8 (100)	18 (78)	10 (63)	13 (93)	
Partial remission	0 (0)	0 (0)	0 (0)	1 (6)	0 (0)	
Relapse	0 (0)	0 (0)	1 (4)	0 (0)	0 (0)	

*Note:* The values are represented as counts and percentages within columns, *n* (%). A *p* value <0.05 is deemed significant.

#### 3.6.2 Association of Age and WT Types and Progression

The association between age and the different WT types (Table 3) showed a statistically significant difference between the age groups and WT extension (*p* = 0.003). Metastatic forms were more frequent in patients over 2 years of age, with 20 cases in total. In detail, 57% of metastatic forms occurred in patients aged between 2 and 5 years, and 38% of metastases manifested in patients aged over 6 years. Likewise, age was associated to WT overall stage (*p* = 0.006). Indeed, 63% of patients less than 2 years of age were in Stage II, and 66% of Stage III cases occurred in the age group between 2 and 5 years. Similarly, age was associated with WT SIOP histology risk‐grades (*p* = 0.042). All patients over 6 years of age had an intermediate or high‐risk WT, with proportions of 60% and 40%, respectively.

**TABLE 3:** Association between age and the different stages and types.

	<2 years	[2, 5 years]	≥ 6 years	Total	*p*
WT extension					0.003
Localised	21 (95)	35 (75)	7 (47)	63	
Metastatic	1 (5)	12 (25)	8 (53)	21	
Total	22	47	15	84	
Overall stage (*n* = 74)					0.006
Stage I	2 (12)	9 (21)	2 (13)	13	
Stage II	10 (63)	9 (21)	1 (7)	20	
Stage III	3 (19)	14 (33)	4 (27)	21	
Stage IV	1 (6)	11 (25)	8 (53)	20	
Total	16	43	15	74	
SIOP histology risk grades					0.042
Low	2 (12)	0 (0)	0 (0)	2	
Intermediate	13 (76)	33 (73)	9 (60)	55	
High	2 (12)	12 (27)	6 (40)	20	
Total	17	45	15	78	
Progress after 5 years from nephrectomy					0.375
Death	6 (27)	10 (21)	3 (20)	19	
Complete remission	14 (64)	37 (79)	12 (80)	63	
Partial remission	1 (4)	0 (0)	0 (0)	1	
Relapse	1 (4)	0 (0)	0 (0)	1	
Total	22	47 (100)	15 (100)	84	

*Note:* The values are represented as counts and percentages within columns, *n* (%). A *p* value <0.05 is deemed significant.

#### 3.6.3 Association of WT Stages and Clinical Presentation

There was no statistically significant association between WT stages and clinical signs (Table 4), except for IVC tumour thrombus, which was strongly correlated with WT stage (*p* = 0.002), as 78% of cases with IVC tumour thrombus were Stage IV.

**TABLE 4:** Correlation between unilateral WT stages and clinical presentation (*N* = 74).

	Stage I (*N* = 13)	Stage II (*N* = 20)	Stage III (*N* = 21)	Stage IV (*N* = 20)	*p*
Abdominal mass	12 (17)	19 (27)	20 (28)	20 (28)	0.715
Gross haematuria	2 (17)	2 (17)	4 (33)	4 (33)	0.823
Microscopic haematuria	0 (0)	1 (50)	0 (0)	1 (50)	0.626
Arterial hypertension	1 (33)	1 (33)	0 (0)	1 (33)	0.700
Anaemia	7 (19)	10 (27)	13 (35)	7 (19)	0.268
IVC tumour thrombus	0 (0)	0 (0)	2 (22)	7 (78)	0.002
Malformation	1 (14)	4 (57)	2 (29)	0 (0)	0.193
Tumour rupture	0 (0)	1 (10)	5 (45)	5 (45)	0.080
Effusion	1 (7)	4 (28)	5 (36)	4 (29)	0.694

*Note:* The values are represented as counts and percentages within columns; *n* (%). A *p* value <0.05 is deemed significant.

## 4 Discussion

A thorough understanding of the clinical features of WT is essential for enhancing its treatment and care. This article provides a comprehensive analysis of the aforementioned traits, evaluating their correlation with histotypes, age and WT stages. Furthermore, we underlined the pivotal significance of the SIOP protocol in the management of WT, highlighting its feasibility in a middle‐income country like Morocco.

WT clinical features usually manifest as non‐specific symptoms such as abdominal pain (30%–40%), a palpable abdominal mass, hypertension (25%, normalised after nephrectomy), gross haematuria (12%–25%), unexplained fever, anorexia and weight loss (in 10% of cases) [1, 6]. Other features include urinary tract infections, varicocele, hypotension, anaemia and hypercalcemia. If the patient has lung metastases, dyspnoea or tachypnoea may occur [1]. It is important to note that these symptoms can often be confused with other common paediatric conditions, such as neuroblastoma, which can lead to a delay in diagnosis [12, 15]. However, the presence of an abdominal mass is often an early warning sign that needs to be thoroughly investigated by clinical examinations.

Our findings align with previous published clinical studies. In the Moroccan study conducted by Madani et al. [16], the median age was 36 months, which matches our results. Remarkably, one of our patients was older than 10 years, which is considered a rare occurrence of WT. A similar Moroccan case was reported by Cherraqi et al. [17] in a 14‐year‐old child. Both cases presented with an abdominal mass, abdominal pain, haematuria and a decline in general condition over 1 month. Furthermore, Madani et al. [16] noted that the abdominal mass is the most frequent clinical sign in 95% of cases, which is consistent with our findings of 96%. However, they reported a 1:1 sex ratio, whereas in our series, we observed a female predominance with a male‐to‐female sex ratio of 0.79.

As reported in the literature [1, 6], bilateral tumours account for approximately 5%–10% of cases (6% in our series) and are more commonly seen in female patients (4/5 metastatic cases in our series). In rare cases, an extra‐renal form of WT may occur. The tumour is located either in the retroperitoneal space or in the area of the small pelvis, in particular the inguinal canal, the female reproductive organs and the testicles [2, 7].

It is noteworthy that specific high‐risk populations require vigilant monitoring, including children with hemihypertrophy, aniridia or BWS, which is an overgrowth syndrome [18]. WT is linked to congenital abnormalities in 10%–13% of cases [2, 19], and our findings align with this frequency at 12%. The likelihood of a patient with BWS developing WT is up to 8% [20]. Within our study population, we identified a case of BWS associated with bilateral WT in a 30‐month‐old female who presented with an abdominal mass, abdominal pain, gross haematuria, iron‐deficiency anaemia, fever, vomiting, weight loss and IVC tumour thrombus. Similar cases have been reported in the literature, as demonstrated in the studies conducted by Hailu et al., Wolfe et al. and Bachmann et al. [21, 22, 23].

The occurrence of IVC tumour thrombus was higher in our cohort (14%) than what is typically reported in the literature (4%–10%) [24]. Elayadi et al. [14] reported a frequency of 7.6% of cases, predominantly in advanced WT stages, particularly Stage IV, with a frequency of 49%. These findings align with our results, as IVC tumour thrombus strongly correlated with WT stage (*p* = 0.002), with 78% of cases with IVC tumour thrombus being Stage IV. The elevated rate of IVC tumour thrombus in our cohort may be attributed to delays in diagnosis and treatment, with a median diagnosis delay of 50 [11–60] days in patients with IVC tumour thrombus. Contributing factors to these delays might include limited healthcare access, lack of awareness about WT signs and symptoms, financial constraints affecting timely medical consultations and the absence of paediatric oncology units in certain regions.

In our cohort, 33 patients (43%) were at local Stage III, consistent with findings from Moroccan and Egyptian studies by Madani et al. [16] and Elayadi et al. [14], respectively, where 44% and 43.1% were in Stage III, respectively. However, the Moroccan study by Rais et al. reported 54% of cases in Stage III. European cohorts estimated only 20.6% of cases in Stage III [25]. This discrepancy may also stem from delayed diagnosis and limited oncology units availability. Notably, only five Moroccan regions have a dedicated oncology unit among the 12 regions [10].

Our analysis explored the relationship between WT histotypes, various WT features and WT progression. Metastasis (Stage IV) was more prevalent in older age groups, potentially due to delayed diagnosis. We found a statistically significant correlation between histotype and sex and tumour localisation, with *p* values of 0.040 and 0.013, respectively, though no significant correlation with WT progression or relapse was observed. In contrast, Fukuzawa et al. indicated that all relapsed cases were blastemal‐predominant, which strongly suggests its role as relapse indicator, with a *p* value of 0.0147 [26]. Notably, in their cohort, only six out of 58 cases experienced relapse (i.e., 10.3%). However, it is known that WT relapses occur in 15% of cases [27, 28]. In the Moroccan study conducted by Rais et al., after a median follow‐up of 54.8 months [20–79], they noted a complete recovery in 61.5% of cases and relapse in 13.5% of cases, with disease progression in 15.4% [29]. In the Moroccan study by Madani et al., after a median follow‐up of 70 months, approximately 65% of patients achieved complete remission, three patients (4%) died due to treatment‐related toxicity and 13 patients (15%) relapsed [16]. Comparing to our cohort, we observed a slight improvement in the complete remission rate, with 75% of patients achieving complete remission. However, the relapse rate during the follow‐up period between 2014 and 2023 aligns with literature data, with 15% of cases relapsed. Interestingly, one of our cases relapsed after 7 years of post‐nephrectomy. This is likely considered a second tumour.

In a multicentric study in sub‐Saharan Africa, the primary cause of treatment failure was treatment abandonment, often due to the financial burdens [30]. This was not evident in our cohort, as all patients completed treatment, except for those who died during the course.

The importance of thoroughly analysing the various clinical and histological characteristics of WT lies in the accurate tumour staging and the proper risk stratification [31], which is the process of categorising patients into different risk groups based on factors such as tumour stage, histological subtype, age, response to treatment and genetic characteristics. This helps personalise treatment to maximise cure rates while minimising side effects and then to decrease the risk of recurrence. According to SIOP histology risk grades, patients are classified into low, intermediate, or high risk and treatment is tailored accordingly [28]. This is especially critical as WT is a challenging cancer to treat in developing countries, where the demand for a wide range of expertise, including imaging, surgery and pathology, together with a multidisciplinary approach. Thus, the employment of standardised treatment approaches that adopt such strategy, as the SIOP protocol, plays an essential role in improving WT outcomes [32, 33]. In African countries, the implementation of the SIOP protocol improved the 2‐year event‐free survival from 46.7% to 77.4% [34]. In a report published in 2022, comparing outcomes of the SIOP protocol of 3176 eligible patients with unilateral WT from 24 countries, a 5‐year overall survival rate of 93% was observed for the entire cohort [25].

As with any retrospective study, we encountered few limitations related to the examination of medical records, in particular the lack of specific details regarding certain biological and radiological parameters, as well as the factors contributing to delays in medical consultations. Therefore, we advocate adoption of a comprehensive patient follow‐up template to ensure the collection and documentation of all relevant patient information, which can be invaluable for ongoing patient monitoring and future clinical research. Another limitation is the restricted timeframe and the relatively small sample size, given the single‐centre design of our study. Furthermore, the scarcity of published Moroccan case series studies presents a challenge, as we relied on a limited number for comparison. Notably, many thesis studies, although rich in valuable research findings, remain unpublished. This underscores the need to publish such studies to enrich the Moroccan literature, thereby enhance the comprehensiveness and robustness of future analyses in WT research.

## 5 Conclusions

These findings are encouraging for a developing country. However, the elevated rates of Stages III and IVC thrombus in this series are still high, primarily attributed to delays in diagnosis and treatment and the limited number of paediatric haematology and oncology units at the time of the study. Nevertheless, further multicentric research is warranted to enrich Moroccan data and establish a national register for WT cases.

The therapy of WT involves a collaborative approach that integrates a meticulous clinical assessment, advanced imaging examinations, accurate histopathological analysis and standardised treatment protocols. Consequently, the post‐operative prognosis is favourable in the majority of cases, making WT a paradigm of successful therapeutic interventions and comprehensive interdisciplinary treatment. Ongoing advances in research and understanding WT aetiology, clinical features and mechanisms is dependent on effective risk stratification. This allows for enabling the prescription of the most appropriate and least invasive treatment for improving short‐ and long‐term outcomes while reducing the risk of relapse.

## Author Contributions

**Sara Benlhachemi:** conceptualization, writing – original draft, methodology, data curation, formal analysis. **Mohammed Khattab:** validation, writing – review and editing. **Kenza Hattoufi:** formal analysis, methodology. **Redouane Abouqal:** methodology, validation. **Saber Boutayeb:** writing – review and editing, validation, methodology. **Elmostafa El Fahime:** writing – review and editing, validation, supervision.

## Ethics Statement

Ethics approval was granted by the Ethics Committee for Biomedical Research of the faculty of Medicine and Pharmacy, Mohamed V University in Rabat, Morocco, under reference number CERB 12/22.

## Conflicts of Interest

The authors declare no conflicts of interest.

## Supporting information


**Supporting Information S1.** STROBE checklist for observational studies (DOCX 30 ko).

## Data Availability

All patient data have been uploaded to Mendeley Data and can be accessed using the identification number: 10.17632/zdhc7fyzr3.1. The dataset is available at the following URL: https://data.mendeley.com/datasets/zdhc7fyzr3/1. For further inquiries, please contact the corresponding author.
